# Citizen Health Science: Foundations of a New Data Science Arena

**DOI:** 10.23889/ijpds.v4i1.1074

**Published:** 2019-09-26

**Authors:** TA Walls, A Coria, SR Forkus

**Affiliations:** 1 University of Rhode Island, Department of Psychology, Chafee Hall, 142 Flagg Road, Kingston, RI 02881

**Keywords:** Citizen health science, single-case designs, single-subject studies, idiographic research, self-experimentation

## Abstract

Citizen scientists with health interests have rapidly increased efforts to conduct their own health studies on themselves and in their communities, giving rise to a new transdisciplinary field of citizen health science. This science leverages long-standing traditions of single case or N-of-1 studies in psychology and also finds influential roots in the history of self-experimentation in health and medicine. These studies frequently incorporate new digital tools such as smartphone tracking and many other mobile health or “mHealth” devices. Citizen health scientists also tend to operate in social networks of people working to maintain or improve their health, increasing the complexity and richness of opportunities tied to this new platform. Population data scientists are well-positioned to seek new ways to derive scientific inferences from data generated in citizen health science projects. This paper provides an overview of citizen health science for population data scientists, including basic definitions, historical foundations, current challenges and opportunities, and future directions.

## Introduction

In this paper, we characterize key tenets of a newly emerging transdisciplinary field of *citizen health science (CHS)*, with special attention to the opportunities and challenges it presents for population data science. We begin by briefly chronicling what this new field involves. We then review some important origins in the single case or N-of-1 tradition in psychology and in relevant aspects of the practice and science of self-experimentation. We further consider a number of approaches and perspectives about individual and population-level inference, and opportunities to advance theory of science, research design and inference in this arena. We conclude with a general overview of key aspects of the growing field of citizen health science.

Our presentation is aimed at advances in scientific approach, theory and methods, although we do mention some empirical studies. We are health psychologists working from diverse disciplinary backgrounds to highlight some unique and important vantage points on population data science. It is our intent that this article will bear relevance to and add value for a range of population data scientists, health researchers and citizen scientists with interests in health inquiry and citizen engagement.

## The field of Citizen Health Science

Recent societal and scientific trends have led to a new scientific platform, one led by citizens, communities and scientists working in service of health goals that people enact and pursue for themselves. Other fields, such as wildlife management, have long had a citizen scientist component, most often comprised of citizen enthusiasts serving as volunteers for wildlife tracking projects or institutionally-led monitoring needs [1—3]. Health science has lagged far behind in adopting a citizen-based approach to health. For most of history and in most cultures, humans have viewed their health as predestined, as dictated by divine forces, predicated by cultural presets, at the discretion of a selected individual such as a priest or shaman, and/or as a set of conditions to be managed by medical science, medical care facilities and industry. The bulk of health science and care has kept patients, and certainly participants in scientific trials, in a relatively disempowered and passive position. This has only lessened slightly with the broad proliferation of homeopathic and natural remedies, changes in medical education, the influence of patient-centered care, and wider access to medical information from the internet. The idea of citizens working in service of their own health through genuine and integral participation in science has arrived on the scene late and is only now starting to gain some support among traditional health scientists.

We are now at the brink of embracing new forms of heath science that prominently feature the interests, roles and stakes of citizens themselves. Several forces have led citizens to study and take action to affect their own health and well-being. These forces include a long tradition in self-experimentation in medicine, the proliferation of self-tracking as a cultural feature, increased education in the general population in support of systematic inquiry, rapid and broad expansion of ambulatory digital sensors, processors, memory and interactive interfaces, a broad and fast mobile grid, vast increases in cloud computing storage and processing capabilities, and increased interest in and availability of societal networking especially in on-line platforms. Most recently, the independent initiative of citizens acting on their own behalf to pursue scientific inquiry for their own benefit and/or in support of family, peers and communities has also gained momentum. These forces are now accelerated both by industries seeking to develop new products and enterprises associated with citizen health science, and by increasing interest in the scientific community in collaborating with citizen health scientists.

Some efforts to define the boundaries of this new space are warranted. CHS is a new transdisciplinary arena focused on questions raised by citizens and pursued scientifically by citizens on behalf of themselves and their networks. This does not preclude scientists from raising questions of concern to a person or community, but the science features citizens who are aware of the questions, engaged in finding answers through tracking their own health, and ready to make use of findings to affect their well-being or survival. CHS involves the initiative of citizens with varying levels of scientific preparation to collect data on their own health and to act of their own accord to change outcomes.

The development of CHS challenges widely accepted assumptions of measurement objectivity, runs counter to traditional approaches to achieving internal and external validity, and involves new change-ups of currently prevailing power structures, leadership norms, funding streams, implementation, and dissemination of results. As in any new science that challenges traditional norms, many criticisms can be levied about threats to scientific validity. We do not strive to mount a defense around how the field is grappling with its scientific challenges in this paper; that is simply not our goal. In fact, we think that many of these issues may take decades for the field as a whole to work through. Instead, we are optimistically focused on the opportunities presented in the field and we contemplate some of the challenges openly. We are very excited about the collaborative space that this emerging field offers and are interested in how some of the challenges can be addressed.

We also strive to share relevant traditions from psychology that are likely to help build the scientific foundations of CHS. We follow this by laying out central themes of the emerging field to provide some structure for population data scientists to consider CHS studies. In any new area of science, it is important to consider foundational sources for scientific inquiry. Some kindred activities have been pursued in psychology and medicine for many decades. We consider briefly these foundations with emphasis on data science relevant issues before moving on to some key inferential methods and a deeper consideration of the emerging CHS field.

## The N-of-1 tradition in psychology and behavioural health

Single subject research can be seen as a logical and historical antecedent to citizen-centered research. In this section, we focus on a) how motivations of several pioneers in early psychological theory were aligned in having an N-of-1 orientation, and b) an active recent movement toward *idiographic* research that has created a new family of research and inferential techniques.

### Early N-of-1 orientations in psychological science

The field of psychology leverages a long tradition of theory, epistemology, and research in consideration of the single case. The founder of the field, Wilhelm Wundt, used informal interviews about introspection as a method of learning about a research participant’s experience in an experiment as his central lens on the psyche [4]. William James’s studies on memory utilized individual cases; in one famous case, he attempted to increase memory through memorization of Victor Hugo stanzas [5]. James Baldwin’s ideas involved a single person’s trajectory and an ‘inductive’ psychology that focused on introspection [6]. Titchener saw introspection as a first-person approach to research, whereas behaviourism became the dominating form of psychology, shifting toward a third person approach to research [7]. Other intrapsychic ideas offered by Freud, Jung and others were inherently person-specific.

With behavioirism and the availability of group experiments and inferential statistics involving group means and variances, much of the field turned to experiments on groups of subjects. Measures of central tendency for the group as a whole and the individual human experience were then routinely conflated, resulting in *ad hoc* generalizations like, ‘people do/feel/think this…’, based on a group mean. Variability among subjects was most often set aside in an error term, used in significance tests, and viewed as measurement error.

As the grip of behaviourism relaxed beginning in the 1980s, the importance of individual differences again came into view in a renewed focus on the processes and feats of a single person. Several attempts to return to a person-oriented viewpoint have arisen in recent scholarship, tying back centrally to the work of Allport [8], who brought renewed focus to the work of Windelband [9]. In this work, the distinction of *idiographic* (single person perspective) versus *nomothetic* (group study perspective) has remained in the fore of theoretical dialogue on inference in psychology to this day [10—11]. In a word, a fundamental need in psychology is to recognize the features and functions of an individual—that each person’s uniqueness must be characterized and respected, while also recognizing an individual’s membership in the species and in specific group contexts. Although we have made the case here that psychology has always incorporated a single-subject orientation, it is the renewed focus on idiographic science that underpins the best contemporary epistemology for single subject research. 

### Population data science aspects of the N-of-1 perspective

Recent theoretical scholarship on inference involving N-of-1 studies takes up several fascinating topics. Key contributions to the methodological literature involving single subject research have been consolidated periodically, through the mid-20^th^ century by Kratochwill [12] and by many other researchers since then, in both qualitative and quantitative areas [13]. Our focus is selective, with attention to some intriguing ideas for population data science. First, Valsiner argues that instead of generalizing from ‘sample to population,’ generalization from the first single instance would be reaffirmed through N-of-1 replications of “single cases” [14]. The generalization then identifies the context or experience of each single case that will likely hold true for the next single case as well.

In complementary work, Molenaar [10] has developed a suite of arguments around the necessity of considering intra-individual variation prominently in psychology. His central argument is that psychological processes are non-ergodic; informally, one slice of data from a person cannot simply be substituted into a data stream of all people and vice versa. This idea is consistent with Valsiner’s assertion that averaging across a heterogeneous class is not sufficient [14]. In order to obtain results from a non-ergodic process that will apply to the individual, the analysis should be based on intra-individual variation, rather than inter-individual variation. This concept is similar to Valsiner’s support for a method based on the generalization from the single instance, to the single case, and then to a generic model [14]. Molenaar [10] provides a more detailed statement on this progression, that by conducting subject-specific data analysis conclusions can be generalized to the population level by obtaining parameter estimates based on the subject-specific analysis of intra-individual variation. Then analyses are conducted on inter-individual variation using these parameter estimates. Hence, both Molenaar [10] and Valsiner [14] suggest that nomothetic knowledge about idiographic processes can be obtained from subject-specific data analysis, by aggregating inferences from instances or slices contributed by each person into a single analysis.

Focal consideration of these individuals may also have important implications for behavioural change efforts. Large-scale studies generalize to a specific population from a small sample of the population of interest. Sample-to-population inferences based on an average score may not be the best representation of the population as a whole. For example, in the work by McDonald and colleagues [15], it was found that while previous studies have suggested that average physical activity declines for individuals after they retire, a series of N-of-1 studies in natural settings revealed that this was not true in many of the cases. In fact, many individuals found themselves having more free time, and were actually more physically active as a result of retirement. To further stress the importance of rejecting a “one size fits all” ideology when it comes to health interventions for the elderly, McDonald and colleagues [15] suggested that individual differences must be taken into consideration to maximize the effectiveness of behavioural interventions.

In keeping with this idea, Hamaker [16] points out that the application of time series analysis (current observation can be predicted from previous) involves generalizing from the single instance to the next ‘similar’ instance. In time series analysis, a relationship between each observation period is assumed, and the prior is believed to predict the subsequent. This implies that a form of generalization is possible from the single instance. She reiterates that information about general laws is not always achieved by studying large representative samples, therefore, averaging across individuals may not be representative of the general population and general laws could be falsely applied to the individual. She advocates for studying individuals over time by using intensive sampling methods over time to observe processes in real-time and analyze the data at the level that it occurs, a recurrent theme in our laboratory’s scholarship as well [11,16—18]. She emphasizes that highly controlled laboratory experiments do not always translate effectively to natural settings, that aggregating single-subject data upwardly can lead to flawed inferences, and she strenuously makes the point that this is insufficient use of valuable single subject data from a person-specific stance—that the information obtained from the aggregate across individuals does not necessarily inform what is actually occurring. Generalizing from information aggregated across individuals to the population may result in assuming false conclusions. In a valuable example, Hamaker [16] describes the relationship between the number of typos and typing speed. At the population level there is a negative relationship, the faster the typing speed, the fewer typos made. However, for any given person, concluding that typing faster will usually result in fewer typos would be false because at the within-person level, there is most frequently a positive relationship, namely, the faster the typing speed, the more typos made.

**Figure 1: Traditional Longitudinal Population Studies versus N-of-1:**
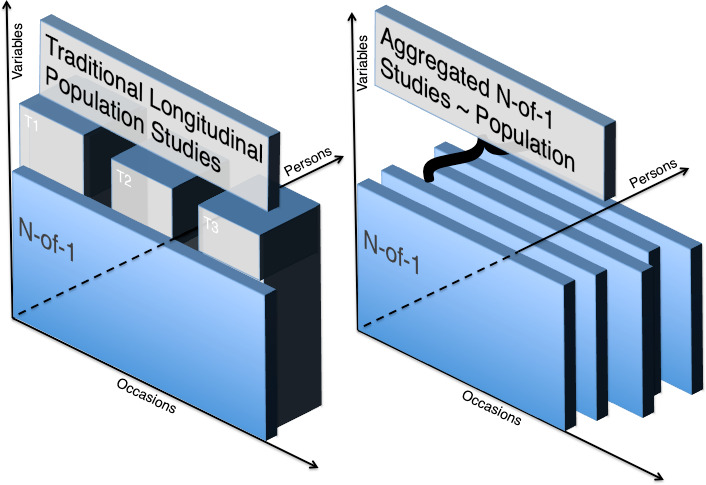


These ideas are compatible with Valsiner’s proposal that we consider independent slices of time as unique, but they are also not compatible in that any aggregation would violate stronger assumptions of temporal and personal uniqueness in his rendering [14]. For our purposes, both presentations are concerned with proper inference to be derived from person-specific data, which is a fundamental need in CHS. In Figure 1, below, we characterize these themes in overview, meaning we are not attempting to depict any particular N-of-1 methodologist’s ideas, but rather to juxtapose potential inferential platforms for contemporary N-of-1 studies with traditional cross-sectional or cohort studies.

Note that in the left panel, N-of-1 studies are limited to inferences about the evolution of one subject, with a selection of variables and, frequently, strong attention to individual evolution over time. By contrast, traditional cohort studies are usually rendered as successive cross-sections, with attention to group means or trends, typically over regularly spaced time windows. In the right panel, consider that opportunities in modern N-of-1 studies lie in considering new aggregates, richer ones built from the rich within-person inferences considered in traditional N-of-1 studies; ‘multiple slices’ if you will. Note that modern studies do not always employ the same starting point, as shown by offset slices in the right panel. These may be tied to recruitment, intervention points, ages, or other time-centering variables. Methods for contemporary person-oriented studies are considered in somewhat greater depth below. Note that N-of-1 studies may be employed in the formative time *en route* to a cohort study or randomized controlled clinical trial. This is certainly a good use of N-of-1 study findings; however, the opportunity to harvest findings via new techniques applied to aggregated N-of-1 studies suggests that much greater opportunities could be realized.

### Technology and Data Science

It is important to share that many modern N-of-1 studies rest on robust technology platforms. Recent contributions on the use of technology in the study of the individual have been consolidated nicely by Mehl and colleagues [19]. A subdiscipline for the analysis of the rich data emerging from technology-based studies emerged in the early 2000s with both technical and theoretical contributions dating far back into the 20th century [11,17—18]. This subdiscipline is now constituent to the high dimensional or big data trends that have emerged across technical fields, though it is distinct in its heavy focus on longitudinal measurement and inference. Practitioner guides for deploying person-oriented studies in various areas of psychology have emerged frequently [20—21], even with specialized intensive longitudinal texts for data analysis [22—23]. An active peer-reviewed literature for theoretical [10, 14, 24], epistemological [16], substantive (peer-reviewed journal contributions), and methodological topics in quantitative psychology journals continues to expand, increasingly moving the N-of-1 approach into the mainstream (See, for example, 25). Whereas software and training in these approaches was previously available sporadically from diverse sources, increasingly these are available in routine formats as workshops, as part of regular graduate-level curricula, and in mainstream and widely available online formats (e.g., 26). On-line communities specifically for the study of the single case remain a bit limited, but these are also increasing as psychological researchers interact with citizens, patients, policy makers, other scientists in the diverse and vibrant arena of self-trackers that have emerged [27]. For population data scientists, this means that the digital record of these studies is often preserved, providing a platform for retrospective or prospective analysis.

### The N-of-1 perspective in emerging precision health studies

Single subject research is also becoming more relevant in health contexts, as medical professionals are moving toward creating more personalized treatments, as opposed to adhering strictly to a traditional public health/epidemiologic format [28—30]. According to the work of Van Ness, Murphy, and Ali [29], this shift in healthcare, known as Complementary and Alternative Medicine (CAM), may be due to the fact that previous health care systems have failed to recognize individual differences and vital needs, which could cause detrimental and tragic outcomes. One of many emerging examples of the N-of-1 focus being beneficial for the individual is described in the work by Xie and Yu [30], in which N-of-1 was used to determine a “perfect” dosage of a particular drug. They were able to optimize the personal benefits of a certain treatment by analyzing the effects of a particular drug, which could vary drastically between person to person, and by using the individual as their own control in the experiment [30]. This ‘tailored healthcare’ is also discussed in the work by Ricciardi and Boccia [28] as “precision medicine”. In considering how false conclusions can be drawn from aggregating across individuals to populations, a more person-specific strategy is necessary in health care contexts to ensure the efficacy of treatment.

N-of-1 and citizen science contributions could also be applied to health care via repositories, as information obtained through these methods (e.g., data collected through wearables) could be aggregated into repositories to allow researchers, practitioners, and citizen scientists access to information to better understand the progression and/or remission of certain health problems across diverse individuals. For instance, individual case reports have been informally aggregated (via social media) contributing to our understanding of Type 2 diabetes remission, and findings have recently been empirically supported [31—32]. Specifically, this information has provided valuable insight into how certain lifestyle changes (e.g., weight loss) may lead to diabetes remission; however, there is considerable individual variability in the successfulness of these lifestyle changes on subsequent diabetes remission, thus access to aggregated repositories of individual data could provide greater insight into individual differences, and could be used to tailor interventions and personalized treatments accordingly.

Using N-of-1 and citizen science approaches can provide valuable information, while building on previous methodological approaches, as they make up for the limitations of previous approaches (e.g., loss of individual information at group level, low power of single subjects), while maximizing the benefits (e.g., richer individual information, aggregate of single subject contributions). They could be applied to a wide variety of health care contexts. Individually tailored approaches could have a dramatic impact when it comes to inequality facing the healthcare system today, as well as promoting better public health for certain populations.

In summary, there is a substantial and growing theoretical basis for a focus on single case or N-of-1 research across health sciences. Extensive arguments can be made for increasing this focus based on technical possibilities such as: the need to evolve a theoretical basis for the multivariate nature of behaviour, the need to characterize the regulatory nature of a person as potentially modeled through time series and control systems models, and the opportunity to develop important linkages to adaptive interventions and personalized medicine. Efforts to advance this scholarship have been appearing in the quantitative psychological methods literature for some time, particularly in the areas relating to health and addiction psychology [33—35]. In addition, qualitative research in transferring ethnographic and anthropological approaches to person-specific analysis, has progressed especially in clinical health areas [36—37]. Although we have only provided brief overview and supplied some linkages to this work here, the increasing size of this scholarship reflects renewed interest and advancement of the single case approach over the last decade or so, with particular attention to aspects that bear strongly on emerging CHS.

## Self-experimentation

Consideration in psychology of the single case has the potential for vast importance in emerging population data science scholarship. However, there is a related approach in health and medicine that is of equal importance. We now consider how the *self-experiment* further leverages the tradition of single subject research.

The topic of self-experimentation has been the source of significant scholarly contributions in both the medical and psychological sciences [38]. For a period of time, cases of self-experimentation appeared in empirical literature and were published in early volumes of *American Journal of Psychology* and *Psychological Review* (e.g., 39 & 40, respectively). In psychology, an interesting late 20^th^ century contribution to the idea of single-case research arose in the work on Neuringer, in a seminal paper on the science of self-experimentation [41]. Around the same time, anecdotal self-experiments have been reported in various idiosyncratic outlets, notably those with an applied orientation (e.g. 42—43). This work did not successfully lead the field to embrace the idea of self-experimentation, though extensive efforts by Roberts (for example, 44) in the past decade or so were noteworthy efforts in this direction. It is important to note that historically, isolated cases of self-experimentation preceded the N-of-1 tradition, but its recognition as a format for new contribution to scientific inquiry did not occur until after N-of-1 was recognized as a potentially viable scientific approach [45].

### The advantages of self-experimentation

Neuringer’s seminal paper deserves a careful review given its prescient and still valuable early and guiding contribution [41]. He proposed that the value of self-experimentation can be found in both the process and outcome of conducting research on the self. Engaging in self-experimentation requires the systematic variation of behaviours in order to study the outcomes; this variability in our rather habitual routines may help us to discover solutions to problematic behaviours and present more effective ways of behaving. In addition, self-experimentation has the unique advantage of enabling assessment of covert phenomena; access to these private events can allow for an analysis of the relationship between the private and the public. The ability to access covert information and witness the outcomes of behavioural variations allows individuals to use this information to formulate personally relevant if-then contingency statements; the previously learned behavioural outcomes imply specific outcomes of the repetition of that behaviour (44, 46). Additionally, a necessary aspect of the research process involves maintaining a personal record of the data. Extensive recordkeeping lends itself to creating a working record of an individual’s behavioural history. Documentation of events may have positive implications for future behaviours because the information recorded may provide insight into behavioural patterns and allow for behavioural modification [47—48]. Self-experimentation places an inherent emphasis on the process of knowledge acquisition because individuals are active participants in the research process. As a result, there is proportionately lesser focus solely on the outcomes. Focusing purely on outcomes implies that there is a single solution to a proposed problem. A focus on the process is more reflective of human behaviours and acknowledges the potential solutions in that space. Therefore, much self-experimentation gives individuals the tools to acquire knowledge to inform action, rather than relying only on the results of professional researchers.

In recent years, as technology has advanced, many self-experimenters have turned to tracking their data electronically. For example, smartphones today have a variety of features that are beneficial for recording experimental data, including but not limited to: text messaging, voice recording, internet access, as well as location data from global positioning systems (GPS). These advantages, currently best consolidated by the smartphone, have led to the creation of what is now called mobile health (mHealth). mHealth has made tracking health habits more accessible than ever before, for example, through dedicated apps, in part leading to an increase of self-experimentation among non-scientists looking to improve aspects of their health. In tracking these health behaviours, longitudinal data may be second to none when it comes to availability and salience, therefore leading to better health decisions by individuals and when shared, more accurate health information in population level analysis [17].

In addition to being utilized for everyday data collection of health and well-being, apps can be used on smartphones to produce more sophisticated data, including, apps for skin cancer detection, electronic stethoscopes, as well as blood tester platforms, just to name a few [49]. Analytics of mHealth applications may also aid in testing the effectiveness of particular treatments when it comes to health psychology, with the recording of a baseline, and over the course of treatment, the recorded results of a proposed intervention [17,47]. These types of applications allow the consumer to be more aware of their own health, and health behaviours, and may encourage users to take responsibility for their health through personalized adjustments, thus allowing individuals to take their health into their own hands. Another major benefit of these practices is that these platforms are relatively user-friendly and inclusive, allowing people from all walks of life the benefit of improving their own health provided by these platforms as citizen-science concepts.

Self-experimentation is an area undergoing rapid advances that have been bearing on multiple areas in social science, especially given the increased use of technology in protocols. New communities are serving as platforms for citizen-scientists to reflect on their own experiences and learn from the experiences of others involved in self-experimentation. Specifically, participants are convening to discuss self-relevant data and findings, generate ideas, share methods for modification and improvement, and highlight potential risks and benefits. They are creating networks of trusted individuals working towards similar goals of self-optimization, such as in the case of a closed loop insulin pump and user community developed by Type I diabetes patient [50—51]. While self-tracking through self-experimentation is often associated with recent technological advances, Lupton [52] argues that these practices have made their mark on history in the past, furthering the idea of a human’s instinct to be critical of themselves in order to improve their overall functioning.

Building upon this idea, as portrayed in the study by Dolejšová and Kera [52], citizen-scientists are drawn to self-experimentation to be in better control of their health. In this particular study, self-scientists adopted a liquid diet, or a “soylent diet”. For example, gaining complete dietary control gave the participants better control over all body functions [53]. Similarly, Roberts [44], for example, through the creation of his “Shangri-La Diet”, believed that if you can create the perfect diet through self-experimentation, then you can have a major impact on your health, as well as many other aspects of your life.

Additionally, online self-experimentation groups can offer social benefits by serving as a source of peer support when it comes to new lifestyle choices, such as abstaining from drinking alcohol [46]. Through these online communities, users may choose to share personal qualitative narratives regarding the impact that self-experimentation had on their quality of life [46], or even more intimate details regarding the effect on their health, through distributing personal medical records or self-tracked data [53].

Self-experimentation may have individual-level benefit beyond the exploration of a single topic (or condition). Roberts [44] argued that self-experimental methods help individuals to examine the benefits of long-term self-experimentation because they help to generate new ideas on ways to improve one’s health. His decade of personally relevant self-experimentation illustrates both the effectiveness and the value of this type of methodology in generating new and plausible methods of bettering oneself. Through his personal experimentation, Roberts [44] found that self-experimentation promotes discovery through a self-catalysis process, as the results of one experiment produce a ripple effect of subsequent discoveries generated by thought-provoking findings (i.e., unexpected results). Additional advantages to self-experimentation include that feasibility and simplicity of the application, which allows for greater leniency in the types of hypotheses tested (i.e., implausible ideas), and due to the self-monitoring nature of this form of methodology, there is the ability to measure the outcome across multiple dimensions allowing for a more comprehensive evaluation of the effect. Over the long-term, the upkeep of a behavioural record can foster the development of new and plausible solutions through identification of behavioural patterns that may have otherwise been overlooked. Overall, Roberts [44] illustrated the significance of self-experimentation as a creative contribution to scientific exploration as a means to formulate worthwhile hypotheses and stimulate new scientific ideas for bettering one’s health through different pathways.

Perhaps the field, or society as a whole, was not ready for these ideas at the time when Neuringer and Roberts made their contributions [43]. However, the N-of-1 movement [27], as well as recent work on methods mentioned in our earlier discourse here, certainly attests to greater readiness now. We have not progressed through all of the notions Neuringer [41] laid out and considerable challenges to the development of N-of-1 research remain, including institutional research review boards which may be unfamiliar with the precepts, limited dissemination outlets, and issues of what constitutes scientific validity. Frequently emerging contributions from a still evolving (though fantastically vibrant and diverse) cadre of researchers with expertise in the area continue to enrich the dialogue around the scientific possibilities of self-experimentation [54].

**Figure 2: Experiments with interrruptions (e.g., self-experimental manipulations):**
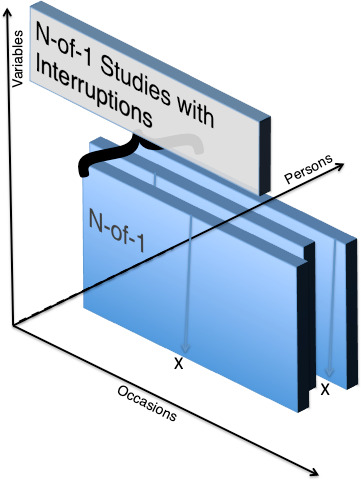


One way to consider developing self-experimentation science further is to consider that every manipulation within an N-of-1 series is a time series interruption. In Figure 2 below, we portray this possibility. Several recent papers have tried to exploit the possibility of new research designs and inferential possibilities tied to this idea [55].

In summary, scientists’ focus on generating knowledge, rather than utilizing it, can lead to a large volume of somewhat underutilized information from an applied perspective. This is especially the case when precepts from research on groups enter into population data science, often forsaking a focus on individual-level concerns. Experiments conducted on and by the individual focus on applying knowledge and thus may increase the likelihood of the results being put into practice because of the pragmatic and personalized process perspective. The mutual benefits of N-of-1 experiments and self-experimentation are key parts of an emerging CHS in this regard. 

### The disadvantages, and liabilities, of self-experimentation

Although the many advantages of the self-experimentation component of CHS are important, the possible drawbacks of this approach cannot be ignored. The work of Dyck and Stewart [38] further investigates that in the past, self-experimenters have been unable to draw clear lines for how far is too far, and that when subjecting one’s own body to scientific research, boundaries may not be so clearly drawn. A human serving as an instrument in measuring changes to itself is often far too challenging a position, considering the dedication needed of the participant for the most accurate results possible and the inherent biases that one carries in relation to one’s own interests, challenges and choices [38]. Often self-experimentation involves the assumption that the human body is machine-like; it will react to a particular stimulant in an expected manner in order to achieve self-optimization. This may not always be the case as our bodies are in fact not machines and may react differently than the body of another person undergoing a similar procedure, or even react differently at a different time [56]. This topic of self-experimentation also reinforces the importance of informed consent, when it comes to the use of human subjects in general. Even when engaging in self-experimentation, it is important that the subject knows all possible outcomes before engaging in a particular study, even at their own hands [38]. Lupton [52] argues that self-experimentation in the form of self-tracking may not always be completely voluntary, and individuals may be exposed to exploitation in order to meet a greater need of a particular study’s political motives. Because of the high risk and respect for individuals’ will in many self-experimentation cases, the possibility of unethical treatment of human subjects in the name of science may be higher than in other human research. Therefore, it is increasingly important to uphold the highest standards of research in this category now and to evolve these standards in the future as this phenomenon grows increasingly popular [45].

In addition to the constant threat of possible physical or psychological harm, self-experimentation also raises questions when it comes to bringing results into mainstream scienceiAlthough our primary interest is more in the health behaviour space rather than in biological experimentation, it is important to mention a trend in ‘biohacking’ given recent notoriety of this area. Biohackers often use unconventional methods in order to fix or improve upon anatomical or physiological structures of the body. As a newer way to look at biomedical health science, biohacking aims to promote the highest level of self-optimization via methods such as gene editing implantable technologies, neodymium magnets, and implantable sensors with Bluetooth capabilities, just to name a few (57). For example, Pelling (58), found that he was able to “hack” an apple to be the biological scaffolding for growing human tissue in the shape of an ear. This type of work shows that biohacking as a science has made great strides, from the early days in which biohackers had to steal and/or obtain scientific instruments from a scientific laboratory (59). Instead, in the case of Pelling (58) and many other biohackers, these scientists have instead turned to using creative methods with unwanted or inexpensive materials in order to find solutions to scientific problems, in ways that may not be traditionally seen as conventional (59). Through his work, Pelling (58) also sought to make his scaffolds available open source, as a way for others to join in, another practice that is often valued by many self-experimenters and biohackers today (57—58).Biohacking, while innovative and exciting as a format to pursue the idea that the human body can be changed using new technologies in order to create optimum functioning, raises new questions in health ethics. For example, with the invention of transplantable devices, biohackers must be weary of safety regarding sterilization as well as cross-contamination between users. Also, as these technologies are relatively new and not always approved by regulatory bodies, little is known of the long term affects that these devices may cause. In addition to these health concerns, biohackers often create online communities to discuss results--questions arise regarding privacy in this type of “open science”, both in terms of personal data privacy and proprietary rights to a device being tested (57). Due to the ambiguous nature of biohacking, certain states of Germany have gone as far as to ultimately outlaw this behaviour, with biohacking being punishable by prison time (60). In the case of CRISPR, a particular tool used by many biohackers in order to conduct particular modifications to a DNA sequence, therefore altering the genes of anything from plant cells to embryos, even amateur biologists are able to use these relatively simplistic tools in order to conduct experiments (61). Although many people may fear the outlandish possibilities that may come along with practices such as these, experts warn that many biohackers have relatively insignificant goals in mind when they start out, for example changing the color of a bacteria, rather than inadvertently altering outcomes for the whole human population. For this reason, it is important to modulate the risk to benefit balance in biohacking. Should it be put to use in a meaningful way, it may become a central tool in searching for cures for diseases that up until now may have been seen as a death sentence (61). Our interest in citizen health science is lesser on biological/medical interventions and greater in the area of health behavioural practices.. For example, the idea that self-experimentation revolves around the use of one’s own body brings inherent skepticism of the biases and possible motives of that individual, or, as in the case of the soylent diet self-experimentation, the effects of possible group bias. In addition to problems underlying natural biases, self-experimentation often does not rely on a systematic schema of research methods in order to gain results, therefore threatening internal validity [53]. On the other hand, Roberts argues that in doing self-experimentation he was actually producing more ecologically valid data then could be produced in a laboratory setting with a strict adherence to scientific protocol, due to the fact that these experiments are often done under much more realistic conditions than the latter [57].

In summary, self-experimentation is an important new area for consideration in population data science, though taken alone, it is one that carries several risks. It can be considered an extension of the single case tradition in that it is more than a description on variables of interest; it inherently involves manipulation or an intervention. It is a field that is deserving of more attention within the data science community, and as an intrinsic advance in idiographic research. If carefully enriched, it may be a feasible option for psychological and behavioural health research, and a key standpoint from which to advance CHS.

## An emerging citizen health science

Psychologists and non-scientists alike have accrued a long history in studying individual (N-of-1) change, and engaging in self-experimentation; arguably this itself is an expression of human free will in modern life. As portrayed in the traditions of the N-of-1 experiments and in our consideration of self-experimentation, it is clear that many people choose to pursue their curiosity by self-tracking and experimenting to control aspects of their health and wellbeing. In keeping, a focus on the needs of the individual has also been growing when it comes to tailored health science, and this is a hallmark feature of CHS, when citizens study themselves or try to act on their own health concerns. However, these foundations are only the starting point for contemporary CHS.

We believe that citizen involvement in health research has much more bounty for health innovation than is seen in the novelty that typically brings it to both scientific and lay outlets. The real innovation here is in a shift of focus, from science being in the minds and hands of the few to the minds and hands of the many. Despite all of its potential limitations, the opportunity for a broad-based participatory science in health is tremendous for science, technology and citizens. What are the key tenets of this emerging science? Based in part on work by Den Broeder, Devilee, Van Oers, Schuit, & Wagemakers, [63], Neuringer [41] and our foregoing analysis of both N-of-1 studies and self-experimentation, we have distilled some defining features of the field, which we are certain will evolve further. They are:

1. *Upward Aggregation.* A focus on the individual as a starting point, moving to groups and possibly whole populations as a later step.
2. *Shared Initiation, leadership, and management.* In this, there is optimistic bias toward the citizen potentially being the source of or guide for all three.
3. *Network Locus.* Studies are frequently embedded in a human network of peers and interested parties, frequently virtual settings. Crowdsourcing is a related concept involving how many minds strive to evolve knowledge on a topic.
4. *Learning Focus.* Studies are frequently aimed at process goals and patient outcomes rather than absolute scientific discovery.
5. *Expanded Scope of Research.* With a large number of participants, studies are able to cover more perspectives than previously deployed in other studies, therefore resulting in more relevant health knowledge.
6. *Open science.* An open sharing format for science helps to reduce the difficulty and burden on citizens in retrieving information, as well as helping them to experience a community of science.
7. *Alternative strategies to achieve validity.* Openness to new methods to derive scientific inferences that attain internal and external validity in ways that are complementary to or widely accepted evidence-based science strategies will support innovation.

Thus, in terms of the N-of-1 tradition and self-experimentation, one manifestation of CHS involves the initiative of citizens with varying levels of scientific preparation to collect data on their own health and to act of their own accord to change outcomes. However, an expanded view includes the possibility that they can take on any and every role in the scientific process, from investigator to collaborator to participant. This is worth further exploration.

In considering CHS we have much to learn from other sciences. Citizen science is by no means exclusive to psychology--it has been employed routinely frequently in ecological research for decades. For example, when the Deepwater Horizon oil spill occurred in 2010, making it the largest single oil spill in American history, experts relied on the actions of citizen scientists to relay what was truly happening in their own communities, based on data that they collected. These citizen scientists found themselves to be more connected to these data as they thought they would be helping their community as a whole, leading to very accurate information on a large scale that could be used to clean the spill and therefore save as much of the wildlife as possible [2]. Similarly, in a citizen health case study that took place in Texas in 2013-2014, citizen scientists were asked to alert scientists when they spot signs of the triatomine bug, or the kissing bug, known for causing the parasitic disease, Chagas disease. By doing so, scientists were able to better understand the geographical distribution and seasonal patterns of this insect in a broader way then would have been possible without citizen-scientists [3]. One of the major similarities between citizen scientists in both of these examples is that when the individual feels a personal connection to the material being studied, they are more likely to dive into the subject wholeheartedly and learn what they can about the subject, all while trying to better their community. Citizen science may be orchestrated differently in health than in ecology, but the former may benefit from technology transfer via review of practices in the latter.

### Some advantages of CHS

**Figure 3: Dimensions for role adoption for CHS studies:**
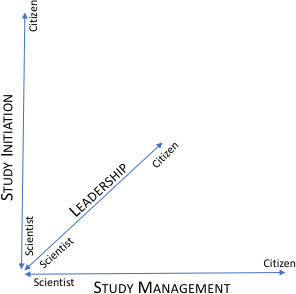


One of the major advantages of CHS is that it enables the citizen participants to serve as the forerunners of their own healthcare. A focus on the needs of the individual emerges, often in contrast to a focus on whole populations, as has guided many health initiatives in the past. For example, according to the work of Woolf and colleagues [64] in order for a healthy lifestyle (diet, exercise, etc.) to be adopted by an individual, efforts for collaborations between different sectors of society should be the main focus, particularly when it comes to health promotion goals. Studies such as this one also suggest an opportunity to integrate health and administrative records with N-of-1 data for a more comprehensive study of health in community and medical context. Another advantage of CHS is the opportunity for collective formation of hypotheses and study designs; with citizen concerns made central to inquiry there is a higher probability of relevant scientific investment for a broader cross-section of stakeholders. For these studies, we believe the relative weights of scientists and citizens involvement need to be evaluated on three dimensions: study initiation, leadership, and management, as shown in Figure 3. Whereas in the past, all three of these roles were typically in the hands of professionally trained researchers, it is necessary to consider ways to change up role adoptions.

For example, it is possible that a citizen with adequate knowledge, access and resources could perform a study with no scientific professionals, although some reports indicate a need to layer responsibilities as citizens gain scientific experience short of full scientific professional identities, preferring the term champion rather than a citizen scientist for community participants [66]. In many areas requiring deep technical or substantive knowledge, it is perhaps somewhat unlikely for most citizen scientists to take on full responsibility, but for consideration it is critical to embrace this possibility as there is a trend in this direction. In particular, note the case of Dana Lewis’s work in a DIY pancreas system [50,51; https://diyps.org/about/dana-lewis/] as a clear and compeling example of a citizen taking the lead. In practice, the weights are likely to be assigned differentially for each study based on its history and needs. Work by Wooley and colleagues [67] similarly emphasizes three overlapping dimensions of citizen-scientist versus scientist-citizen collaborations: engagement, participation and involvement. They sound some warnings about risks to citizens from formal structures, especially in the case of biomedical experimentation, and consider a “social contract” approach at length for its potential merits in clarifying roles, responsibilities, and risks.

Work by Prainsack [68] also considers the inputs of citizen science (CS), from realms ranging from crowdsourcing to social networks, and in consideration of factors such as self-selection or other motivations to participate. Even while these types of citizen science have advanced mostly due to broad-based increases in the availability of technology, it has been suggested that different kinds of citizen science are steeped in traditions going back generations, may operate differently, and manifest themselves differently than others. Prainsack and colleagues describe a range of project courses, from being considered “top down”, with some scientifically trained individuals involved in “bottom up” research initiatives, or strictly citizen-run initiatives without the backing of any research institution. In particular cases, gaming has even been used in CS initiatives, as a way to tackle major problems in a crowd source arena.This particular approach is quite distal from data collection typically employed in population-based research. Finally, it is very possible that the definitions of how data supplied from one person to another can be used for will become more stringent, as is already notable in the European Union’s General Data Protection Regulation (GDPR). This set of laws limits public data uses and increases personal control of data in highly specific ways, especially the kinds of health data that tend to be shared in on-line archives by citizens. However, in order for all of these types of inputs to be accepted as science, there must be a redefining in society of what it means for data to be considered to be scientific, and, in keeping how this new data generated should be evaluated and assessed as a scientific entity in its own right [67—68].

Emerging citizen health science projects are increasing in number, breadth and type. For example, Dana Lewis’s work on a do-it-yourself pancreas represents a citizen biomedical technology intervention that was motivated by the desire for greater safety and improved lifestyle for Type I diabetes patients (see https://diyps.org/). Well-known work by Larry Smarr demonstrated that tracking of his own laboratory tests and medical records enabled him to detect a downturn in his health reflecting incipient Crohn’s symptoms [70,71]. Both of these cases show how self-tracking and trend detection can be used to drive self-care and personalization by citizens themselves. Work discussed in this article in the areas of medical experimentation, N-of-1 science and self-tracking in general all reflect seminal and influential cases of practices supporting a broader citizen health science arena.

### Some potential liabilities

Although the benefits of this trend toward citizen self-science may be numerous, the idea also raises several concerns. For example, when a citizen is engaging in science with a personal stake, indeed their own stake, they will likely bring with them their own inherent biases, which in turn may impact their research results. If citizen scientists report results of nonsystematic inquiry, the scientific community’s credibility may become a problem down the road [60]. This is arguably more likely to occur at the hands of those who are not trained heavily than at the hands of those who are (though both are entirely possible). Another possible liability for citizen science is that it relies heavily on the citizen scientist being inherently accountable for data provided and shared with the scientific community, bringing up a major issue privacy for that individual [65]. This problem may be even more prominent in the world of behavioural and psychological studies, where participants are asked to share some of the more intimate aspects of their lives in the name of science. Another set of risks lies in the possibility of a reductionism of individuals to their cases—overly attending to their personal case to the exclusion of well-established scientific findings and often without sufficient scientific training guiding the thought process. For example, opting to not administer vaccines to children based on nonsystematic personal assessments of their risks could endanger public health in ways that are not consistent with the proposed goals of CHS, which strive for increased well-being through scientific emphasis and engagement of individuals in study of their health concerns. Despite any challenges that this emerging field faces, it is also increasingly important that society does not place overly prohibitive regulations on citizen scientists, as in turn this could lead to a stifling of scientific outcomes and data generation as a whole, while also disempowering citizens of their right to participate in the study of their health, if not of their rights in general [68].

## Discussion

In this paper, we have covered selected themes related to the single case tradition as they inform an emerging CHS. We touched on how early psychologists were largely working from a single case conception, considered how important theoretical and methodological contributions have led to renewed interest in single subject research in psychology, and proposed that self-experimentation has the ability to further advance the single case tradition. In doing so, self-experimentation, along with the long-standing tradition of N-of-1 experiments, have provided key defining precepts of CHS. We provided a summary of Neuringer’s seminal contribution on self-experimentation and substantial subsequent contributions by Roberts, proposing that this work be considered both in relation to past themes of single subject research and to contemporary movements toward idiographic research [41]. Self-experimentation places an inherent emphasis on the individual, thus allowing for the focus of psychological inquiry to return to a more idiographic approach. Additionally, this method has been shown to be a valuable contribution to scientific knowledge through its historical applications, but with recent technological advancements, the capabilities of self-experimentation could be more advantageous than ever before—it is in this context that researchers with data analytic skill sets have the potential in increase both the quality and diversity of data science scholarship in CHS.

Centrally, an increased number of data science-based demonstrations involving technology monitoring in public and private spaces, with N-of-1 tracking, with or without self-experimentation, will form the basis of the emerging transdisciplinary field of CHS. How designs and inferences around these experiments can be refined for participants in many roles and for dissemination in a range scientific and public access outlets, will form precepts and norms of citizen health science. It is in this context that technical contributions from population data scientists will be most influential.

## Acknowledgments

Prof. Emeritus John Stevenson of the Department of Psychology at the University of Rhode Island provided valuable review of early versions of this paper and we thank him for his assistance. Our thoughts on CHS were also enriched by dialogue at the 2016 Quantified Self conference and student participation in an honor’s course on the topic of self-experimentation in citizen science.

## References

[1] Kullenberg C, Kasperowski D. What is citizen science? – a scientometric meta-analysis. Plos One [Internet]. 2016; 11(1): 1-16. Available from: 10.1371/journal.pone.0147152 PMC4713078 26766577

[2] McCormick S. After the cap: risk assessment, citizen science and disaster recovery. ecology and society. 2012;17(4). 10.5751/ES-05263-170431

[3] Curtis-Robles R, Wozniak E, Auckland L, Hamer G, Hamer S. Combining public health education and disease ecology research: using citizen science to assess Chagas disease entomological risk in Texas. PLOS Neglected Tropical Diseases. 2015;9(12):e0004235 10.1371/journal.pntd.0004235 26658425 PMC4687635

[4] Danziger K. Wundt’s psychological experiment in the light of his philosophy of science. Psychological Research. 1980;42 (1-2):109-122 10.1007/BF00308696

[5] James W. The principles of psychology. Read Books Ltd; 1890.

[6] Baldwin J. Handbook of psychology. New York: Henry Holt; 1890.

[7] Titchener E. Experimental psychology: a manual of laboratory practice. London: Macmillan; 1901.

[8] Allport G. Personality: A psychological interpretation. New York: Henry Holt; 1937.

[9] Windelband, W. History and natural science. Theory & Psych. 1998;8(1), 5-22. 10.1177/0959354398081001

[10] Molenaar P.C.M. A Manifesto on psychology as idiographic science: bringing the person back into scientific psychology, this time forever. Measurement: Interdisc Res & Persp. 2004;2(4):201-218. 10.1207/s15366359mea0204_1

[11] Walls T.W. Intensive longitudinal data In: Little, T.D., ed., Oxford Handbook of Quantitative Methods. 2013.

[12] Kratochwill T. Single subject research: strategies for evaluating change. University of Arizona: Academic Press; 2013.

[13] Kazdin A. Single-case research designs. New York: Oxford University Press; 1982.

[14] Valsiner J. Generalization is possible only from a single case (and from a single instance): the value of a personal diary In: Wagoner B, Chaudhary N, Hviid P, ed. by. Integrating experiences: body and mind moving between contexts. Charlotte, NC: Information Age Publishers; 2018 233-244.

[15] McDonald S, O’Brien N, White M, Sniehotta F. Changes in physical activity during the retirement transition: a theory-based, qualitative interview study. Intl J of Beh Nutr and Phys Act.2015;12(1):25. 10.1186/s12966-015-0186-4 PMC4343052 25889481

[16] Hamaker E. Why researchers should think “within-person”: a paradigmatic rationale Handbook of research methods for studying daily life. 2012;43-61.

[17] Smith D, Walls T. mHealth analytics In: Marsch L, Lord S, Dallery J, ed. by. Transforming behavioral health care with technology: the state of the science. New York: Oxford University Press; 2018.

[18] Walls T, Schafer J. Models for intensive longitudinal data. Oxford: Oxford University Press; 2006.

[19] Mehl M, Conner T, Csikszentmihalyi M. Handbook of research methods for studying daily life. New York: Guilford Press; 2012.

[20] Hektner J, Schmidt J, Csikszentmihalyi M. Experience sampling method: Measuring the quality of everyday life. Sage 2007.

[21] Stone A, Shiffman S. Capturing momentary, self-report data: A proposal for reporting guidelines. A of Beh Med. 2002;24(3):236-243. 10.1207/S15324796ABM2403_09 12173681

[22] Bolger N, Laurenceau J. Intensive longitudinal methods. New York: Guilford Press; 2013.

[23] Kazdin A. Single-case research designs. New York, N.Y.: Oxford University Press; 2011.

[24] Silverstein A. An Aristotelian resolution of the idiographic versus nomothetic tension. Amer Psych. 1988;43(6):425-430. 10.1037/0003-066x.43.6.425

[25] Nesselroade J, Molenaar P. Some behavioral science measurement concerns and proposals. Multivar Behav Res. 2016;51(2-3):396-412. 10.1080/00273171.2015.1050481 PMC4990147 27248831

[26] Martella R. Understanding and interpreting educational research. New York, NY: The Guilford Press; 2013.

[27] Quantified Self – Self-knowledge through numbers [Internet]. Quantified Self 2018 [cited 21 January 2016]. Available from: http://quantifiedself.com/

[28] Ricciardi W, Boccia S. New challenges of public health: bringing the future of personalised healthcare into focus. Eur J of Publ Health. 2017;27(suppl_4):36-39. 10.1093/eurpub/ckx164 29028243

[29] Van Ness P, Murphy T, Ali A. Attention to individuals: mixed methods for N-of-1 health care interventions. J of Mixed Meth Res. 2016;11(3):342-354. 10.1177/1558689815623685 PMC5518787 28736512

[30] Xie T, Yu Z. N-of-1 design and its applications to personalized treatment ttudies. Stat in Biosci. 2016;9(2):662-675. 10.1007/s12561-016-9165-9 PMC5711967 29225716

[31] Taylor R, Al-Mrabeh A, Zhyzhneuskaya S, Peters C, Barnes AC, Aribisala BS, et al Remission of human type 2 diabetes requires decrease in liver and pancreas fat content but is dependent upon capacity for β cell recovery. Cell Met [Internet]. (2 8 2018);28(4):667. Available from: https://www.ncbi.nlm.nih.gov/pubmed/30078554 10.1016/j.cmet.2018.08.010 30282047

[32] Lean ME, Leslie WS, Barnes AC, Brosnahan N, Thom G, Mccombie L, et al Primary care-led weight management for remission of type 2 diabetes (DiRECT): an open-label, cluster-randomised trial. The Lancet [Internet]. 2018;391(10120):541-551. Available from: https://www.ncbi.nlm.nih.gov/pubmed/29221645 10.1016/S0140-6736(17)33102-1 29221645

[33] Nandola N, Rivera D.E. A novel model predictive control formulation for hybrid systems with application to adaptive behavioral interventions. In proceedings of the 2010 American Control Conference 2010;6286-6292. 10.1109/ACC.2010.5531515 PMC2935661 20830213

[34] Ramsay J.O. The control of behavioral input-output systems In: Walls T, Schafer J, ed. by. Models for intensive longitudinal data. New York: Oxford University Press; 2006 176-194. 10.1093/acprof:oso/9780195173444.003.0008

[35] Strecher V, Wang C, Derry H, Wildenhaus K, Johnson C. Tailored interventions for multiple risk behaviors. Health Education Research. 2002;17(5):619-626. 10.1093/her/17.5.619 12408206

[36] Reavey P. Visual methods in psychology: using and interpreting images in qualitative research. New York: Routledge; 2012.

[37] Smith J. Qualitative psychology: a practical guide to research methods. London: Sage; 2007.

[38] Dyck E, Stewart L. The uses of humans in experiment. Leiden: Brill Rodopi; 2016.

[39] Lombard W. The effect of fatigue on voluntary muscular contractions. Amer J of Psych. 1890;3(1):24.

[40] Thorndike A. Mental fatigue. Psych Rev. 1900;7(6):547.

[41] Neuringer A. Self-experimentation: a call for change. Behaviorism. 1981;9(1):79-94

[42] Mahoney M. Cognitive behavior modification. Oxford: Ballinger; 1974.

[43] Roberts S, Neuringer A. Self-Experimentation In: Latal K, Perone M, ed. by. Handbook of research methods in human operant behavior. New York: Plenum; 1998. p. 619-655. 10.1007/978-1-4899-1947-2_19

[44] Roberts S. Self-experimentation as a source of new ideas: examples about sleep, mood, health, and weight. Behavioral and Brain Sciences. 2004;27(02):227-262. 10.1017/S0140525X04000068 15595236

[45] Altman L. Who goes first? Los Angeles: University of California Press; 1987.

[46] Carah N, Meurk C, Angus D. Online self-expression and experimentation as ‘reflectivism’: Using text analytics to examine the participatory forum Hello Sunday Morning. Health: An interdisciplinary journal for the social study of health, illness and medicine. 2016;21(2):119-135. 10.1177/1363459315596799 26216897

[47] Dallery J, Cassidy R, Raiff B. Single-case experimental designs to evaluate novel technology-based health interventions. J of Med Internet Res. 2013;15(2):e22 10.2196/jmir.2227 23399668 PMC3636286

[48] Walls T, Barta W, Stawski R, Collyer C, Hofer S. Time-scale dependent longitudinal designs In: Laursen B, Little T, Card N, ed. by. Handbook of developmental research methods. New York: Guilford Press; 2011.

[49] Rothstein M, Willbanks J, Brothers K. Citizen science on your smartphone: an ELSI research agenda. J of Law, Med, and Ethics. 2015;43(4). 10.1111/jlme.12327 26711425

[50] Lewis D. We are not waiting. Presentation presented at Quantified Self Conference, San Diego 2015.

[51] Lewis D. Opening pathways for discovery, research, and innovation in health and healthcare [Internet]. DIYPS.org. 2018 [cited 10 October 2018]. Available from: https://diyps.org/2017/09/15/opening-pathways-for-discovery-research-and-innovation-in-health-and-healthcare/

[52] Lupton D. The quantified self: a sociology of self-tracking. Cambridge: Polity; 2016.

[53] Dolejšová M, Kera D. Soylent Diet Self-Experimentation: Design Challenges in Extreme Citizen Science Projects. Proceedings of the 2017 ACM Conference on computer supported cooperative work and social computing CSCW '17. 2017 10.1145/2998181.2998365

[54] Karkar R, Zia J, Vilardaga R, Mishra SR, Fogarty J, Munson SA, et al A framework for self-experimentation in personalized health. J of the Amer Med Inform Assoc. [Internet]. 2015;23(3):440-448. Available from: https://www.ncbi.nlm.nih.gov/pubmed/26644399 10.1093/jamia/ocv150 PMC6095104 26644399

[55] Lee J, Walker E, Burleson W, Kay M, Buman M, Hekler EB. Self-experimentation for behavior change. Proceedings of the 2017 CHI Conference on Human Factors in Computing Systems - CHI 17 [Internet]. 2017; Available from: http://www.jisoolee.net/publications/2017_CHI_Self_Experimentation.pdf 10.1145/3025453.3026038

[56] Ruckenstein M, Pantzar M. Beyond the Quantified Self: thematic exploration of a dataistic paradigm. New Media & Society. 2016;19(3):401-418 10.1177/1461444815609081

[57] Roberts S. The reception of my self-experimentation. J of Bus Res. 2011;65(7):1060-1066.

[58] Yetisen A. Biohacking. Trends in biotechnology. 2018;36(8):744-747 10.1016/j.tibtech.2018.02.011 29550160

[59] Pelling A. This scientist makes ears out of apples [Internet]. Ted.com. 2018 [cited 10 October 2018]. Available from: https://www.ted.com/talks/andrew_pelling_this_scientist_makes_ears_out_of_apples/up-next

[60] Golinelli S, Ruivenkamp G. Do-it-yourself biology: Action research within the life sciences. Action Res. 2015;14(2):151-167. 10.1177/1476750315586636

[61] Biohackers can boost trust in biology. Nature. 2017;552(7685):291-291. 10.1038/d41586-017-08807-z 29293225

[62] Ledford H. Biohackers gear up for genome editing. Nature. 2015;524(7566):398-399. 10.1038/524398a 26310746

[63] Den Broeder L, Devilee J, Van Oers H, Schuit A, Wagemakers A. Citizen science for public health. Health Prom Intl. 2016 10.1177/1476750315586636 PMC6005099 28011657

[64] Woolf S, Dekker M, Byrne F, Miller W. Citizen-centered health promotion building collaborations to facilitate healthy living. Amer J of Prev Med. 2011;40(1):S38-S47. 10.1016/j.amepre.2010.09.025 21146777

[65] Frankish C, Kwan B, Ratner P, Wharf Higgins J, Larsen C. Challenges of citizen participation in regional health authorities. Social Sci & Med. 2002;54(10):1471-1480. 10.1016/S0277-9536(01)00135-6 12061482

[66] Towson, J. Protocol for implementing the concept of citizen scientists for HealthWise Wales: A national population study. Intl J of Pop Data Sci. 2017;1,106. 10.23889/ijpds.v1i1.125

[67] Wooley JP, McGowan ML, Teare HJA, Victoria Coathup, Fishman JR, Settersten RA, et al Citizen science or scientific citizenship? Disentangling the uses of public engagement rhetoric in national research initiatives. BMC Medical Ethics [Internet]. 2016;17(33):1-17. Available from: 10.1186/s12910-016-0117-1 27260081 PMC4893207

[68] Fiske, A., Del Savio, L., Prainsack, B., Buyx, A. Conceptual and ethical considerations for citizen science in biomedicine In: N. Heyen, S. Dickel and A. Brüninghaus, ed., Personal Health Science. [online] Springer VS, Wiesbaden, pp.195-217. Available at: https://link.springer.com/chapter/10.1007/978-3-658-16428-7_10 2019. 10.1007/978-3-658-16428-7_10

[69] Ragan, S.M. Medicine ignored this insulin problem. Hackers solved it. Neo. Life. [online] https://medium.com/neodotlife/dana-lewis-open-aps-hack-artificial-pancreas-af6ef23a997f 2018.

[70] Bowden, M. The man who saw inside himself. The Atlantic. https://www.theatlantic.com/magazine/archive/2018/03/larry-smarr-the-man-who-saw-inside-himself/550883/ 2018.

[71] Smarr, L. Quantifying your body: A how-to guide from a systems biology perspective. Biotech Journal. 2012;7,980-991. 2012. 10.1002/biot.201100495 22887886

